# Mechanical Properties of Polyethylene Fiber Reinforced Ultra High Performance Concrete (UHPC)

**DOI:** 10.3390/ma15248734

**Published:** 2022-12-07

**Authors:** Xin Zhao, Lei Cai, Xiaohua Ji, Wei Zeng, Jintao Liu

**Affiliations:** 1 School of Civil Engineering and Architecture, Zhejiang University of Science and Technology, Hangzhou 310023, China; 2 College of Civil Engineering and Architecture, Zhejiang University, Hangzhou 310058, China; 3 Zhejiang Construction Investment Group Co., Ltd., Hangzhou 310013, China; 4 College of Civil Engineering, Zhejiang University of Technology, Hangzhou 310023, China

**Keywords:** UHPC, polyethylene fiber, tensile mechanical behavior, optimum fiber

## Abstract

Ultra-high performance concrete (UHPC) is a kind of cement-based material with ultra-high strength, high toughness and excellent durability. However, the tensile strain capacity of UHPC is often below 0.5%, and the mode of single crack failure is the main failure pattern, which limits the development of UHPC. In order to overcome the weakness of the relatively low strain capacity of UHPC, five types of polyethylene (PE) fibers with different geometrical and mechanical parameters (length, diameter and elastic modulus) were added into the matrix, and the corresponding mechanical behavior was investigated. The experimental results showed that the high fiber length and fiber diameter of PE fibers are a benefit for the compressive strength and tensile strength of UHPC. The increase of the fiber diameter and elastic modulus remarkably attributed to the increase in the tensile strain capacity of UHPC. With the increase of the fiber diameter and elastic modulus, the overall energy absorption capacity *G* and the energy absorption capacity of the substrate prior to the softening section *g*_a_ of UHPC were both enhanced. The diameter of PE fiber was the main factor affecting the energy consumption of UHPC. Among the five types of PE fiber, *PF* fiber (*PF* fiber is PF type polyethylene fiber; Fiber length: 15 mm; Fiber diameter: 27 μm; Elastic Modulus: 117 GPa) is the optimal fiber to increase the tensile mechanical behavior of UHPC.

## 1. Introduction

Ultra-high performance concrete (UHPC) is a kind of cement-based material with ultra-high strength, high toughness and excellent durability. The compressive strength of UHPC material is generally 3–16 times that of normal concrete, its tensile strength is more than 5 MPa, and it shows high toughness and fracture energy [1,2,3]. Due to the superior mechanical properties of UHPC, the weight of structural elements can be reduced by more than half while satisfying the same loading requirements [4]. Moreover, the superior mechanical behavior of UHPC gives rise to high energy absorption under dynamic loading, thus making such material suitable for application in bridges, nuclear power plants and military facilities to resist impact, penetration and blast [5,6,7]. 

Incorporation of fibers is vital to improve the crack resistance and fracture toughness of UHPC. Steel fiber is the most common used fiber to improve the toughness of UHPC, and extensive studies have been conducted in this area [2]. The reinforcement effect of steel fiber on the UHPC matrix is influenced by the fiber content, fiber shape, fiber length and aspect ratio [2,3,8]. When the steel fiber content is below 3%, the existing research showed that the strength of UHPC increased at different magnitudes with the increase of steel fiber content under compressive, tensile and flexural loading [3]. However, further increase of fiber content in UHPC led to the decrease of flowability and the presence of fiber agglomerate, resulting in the decline of the mechanical properties of UHPC [3,9,10]. The results of research on the influence of fiber shape on the mechanical properties of UHPC explored by different researchers were not consistent. Some research indicated the deformed fibers, i.e., hook-end fiber, twist fiber and corrugated fiber, are beneficial for the mechanical properties of UHPC compared to the straight fibers [11,12,13], whereas some other researchers reported the opposite results [8,14]. Yoo et al. [15] demonstrated that straight steel fibers exhibited higher fiber numbers per unit volume and a better distribution condition, which may be the reason for the better mechanical properties of UHPC containing straight steel fibers. Moreover, some other studies have been carried out to investigate the influence of steel fiber length and aspect ratio on the mechanical behavior of UHPC. The results showed that steel fiber length and aspect ratio have a positive effect on the tensile and flexural behavior of UHPC [11,16,17], but they have no obvious influence on the compressive behavior [18]. Compared with plain concrete, steel fiber reinforcement can improve the cracking resistance and fracture toughness of UHPC, but the mode of single crack failure is still the main failure pattern; the crack width has not been effectively controlled, and the ultimate strain capacity is still limited [2,3].

In order to improve the strain capacity of UHPC, synthetic fibers with relatively lower elastic modulus and higher aspect ratio (compared with the steel fiber) were adopted. Polyethylene (PE) fiber is one kind of synthetic fiber that has been widely used in strain hardening cementitious composite (SHCC) to achieve strain hardening behavior and multiple crack phenomenon. Due to the bridging effect of PE fiber, SHCC possesses a strain capacity of over 3% and a crack width less than 200 μm [19,20]. Compared with SHCC, UHPC possess a lower water-to-cement binder ratio, lower porosity and higher strength, all of which have wide potential applications in harsh environments [21,22]. However, the shortage of strain capacity was the factor constraint in the development of UHPC. Since PE fibers are expected to be used in UHPC to bridge microcracks, prevent crack propagation and improve ductility, some pioneer studies have been focused on UHPC reinforced with PE fibers [23,24,25,26,27]. The results indicated that the tensile and flexure behavior of composites improved significantly by adding an appropriate amount of PE fiber. However, the compressive strength of UHPC exhibited the opposite result. Yu et al. [25] investigated the influence of PE fiber content on the compressive strength of UHPC, and the results indicated that fiber addition led to a decrease in compressive strength of 9.8–14.3%. Since PE fiber is a hydrophobic, the interfacial zone between the fiber and the matrix is porous [28,29]. In addition, the high aspect ratio of PE fiber may tend to make the fibers agglomerate, causing poor fiber dispersion in composites during mixing [3,30]. These reasons led to the negative effect of PE fibers on the mechanical behavior of composites. The effect of PE fibers on UHPC is also related to fiber content and fiber parameters. Zhang et al. [26] tested the tensile properties of PE fiber-reinforced UHPC containing different fiber content and fiber lengths. The increasing fiber content (1–2%) and fiber lengths (6–18 mm) caused an increase of strain capacity, where the composites with 2% of 18 mm PE fiber exhibited the highest strain. Although some pioneer work has been done on the mechanical properties of PE-reinforced UHPC, a few studies discussed the influence of the morphology and basic parameters of PE fiber on the mechanical properties and energy absorption capacity of UHPC in detail.

In this study, five types of PE fibers distinguished by length, diameter, elastic modulus and tensile strength were used to reinforce UHPC where the fiber content was kept constant, i.e., 2% volume. The effect of the PE fiber type on the static compressive strength, tensile behavior and energy absorption capacity of UHPC were discussed. The optimum PE fiber type was chosen to improve the mechanical properties of UHPC.

## 2. Experimental Program

### 2.1. Raw Materials and Mix Proportions 

The raw materials used in the developed UHPC include ordinary Portland cement 52.5R (OPC, Hangzhou Cement Group Co., Ltd., Hangzhou, China), silica fume (SF), slag, fine silica sand (fineness modulus is 1.5, apparent density is 2350 kg/m^3^) (silica fume, slag and fine silica sand were produced by Zhejiang Zhongzhou Silicon Industry Co., Ltd., Hangzhou, China), superplasticiser (Melflux 4930F, Hangzhou Shibao Building Materials Technology Co., Ltd., Hangzhou, China) and defoamer (German Mingling P803, Hangzhou Shibao Building Materials Technology Co., Ltd., Hangzhou, China). The basic parameters of cement are shown in Table 1. Chemical composition and physical properties of silica fume are shown in Table 2. The S105 grade of granulated blast furnace slag is used, and it is in accordance with the standard of the “Ground granulated blast furnace slag used for cement and concrete” (GB/T 18046-2008).

**Figure 1:**
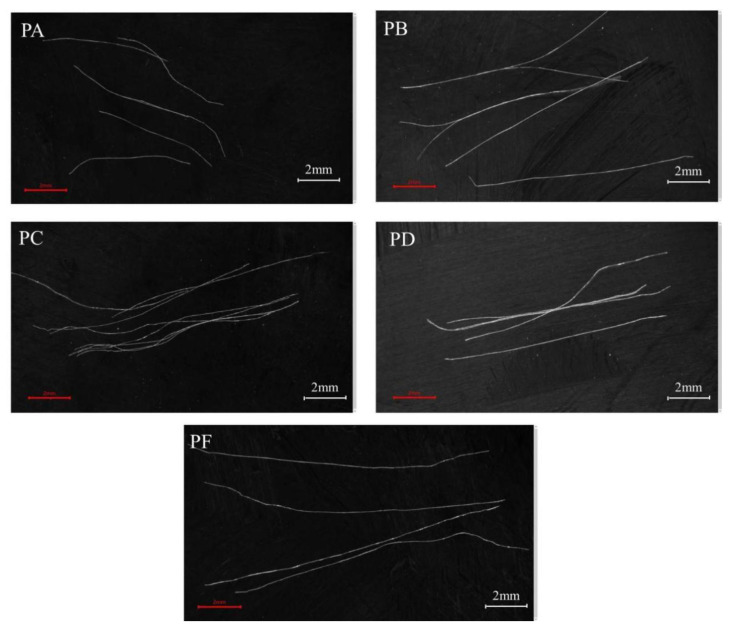
Morphology of PE fiber.

Ultra-high molecular weight polyethylene (PE) fibers with 2% vol. were used in the UHPC specimens. The mix design of the UHPC matrix is given in Table 3. PE fiber has a high modulus of elasticity and fiber strength. There are 5 types of PE fiber, namely *PA* fiber, *PB* fiber, *PC* fiber, *PD* fiber, *PF* fiber (i.e., *PF* fiber is *PF* type polyethylene fiber). The morphology and basic parameters are shown in Figure 1 and Table 4, respectively.

**Figure 2:**
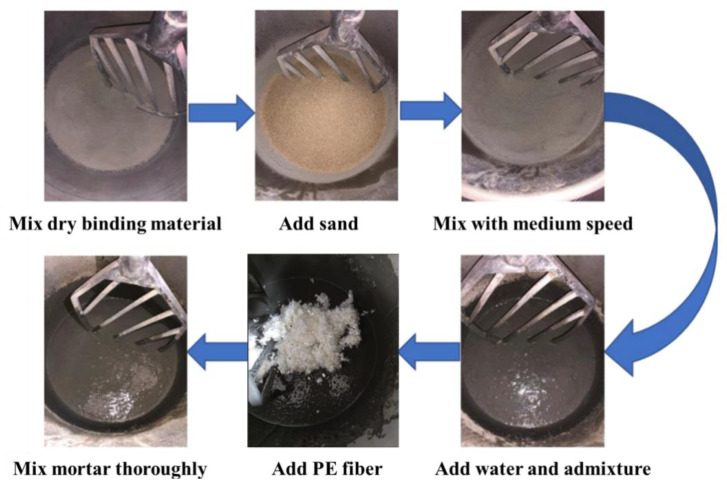
Mixing process of UHPC.

B30 mixer was used in this study for mixing. The mixing speed is divided into low, medium and high grades (65/102/296 r/min). The dry components, including cement, silica fume and slag, were first mixed in the mixer for 3 min on the medium mixing speed. The sand was added into the mixer slowly and mixed for another 2 min. Then the water, superplasticiser and defoamer were added for 5 min of mixing. Finally, the PE fiber was added into the fresh mortar slowly and mixed until the fibers were dispersed uniformly in the matrix without fiber agglomeration. The mixing process of the UHPC is given in Figure 2. The values of the slump test regarding the workability of the fresh UHPC are listed in Table 3. It can be seen that, compared to the plain UHPC (its slump value is 265 mm), the slump values decline gradually with the addition of PE fiber. The slump values of all mixtures are larger than 130 mm.

After mixing, the fresh mixture was poured into the mold and vibrated in the vibrating table. After casting, the plastic film was placed over the specimens to keep them wet. After 24 h, the specimens were de-molded and kept in a standard curing room (temperature 20 ± 2 °C, humidity > 95%) for 28 days.

### 2.2. Test Procedure 

**Figure 3:**
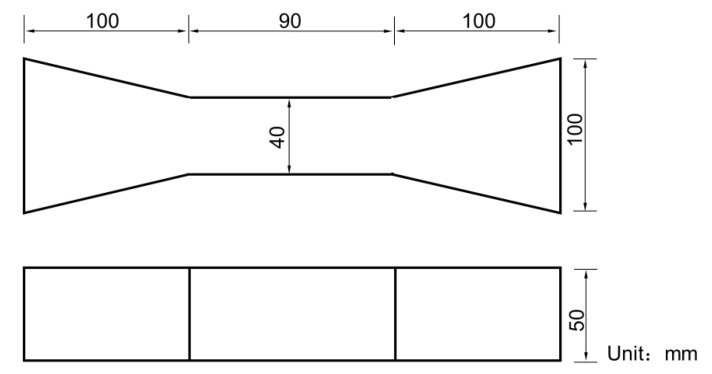
Schematic diagram of the size of the tensile specimen.

A STYE-3000C testing machine was used to test the compressive strength of the UHPC. The size of the compressive specimens was 70.7 × 70.7 × 70.7 mm^3^. The load-controlled experiment was adopted with the loading rate of 2.4 ± 0.2 kN/s. Dog-bone specimens with a total length of 290 mm and cross-sectional dimensions of 40 × 50 mm^2^ in the middle region were used to investigate the effect of strain rate on the tensile behavior of the UHPC in this study, as shown in Figure 3. Six compressive specimens and at least four dog-bone specimens were prepared for each type of sample.

**Figure 4:**
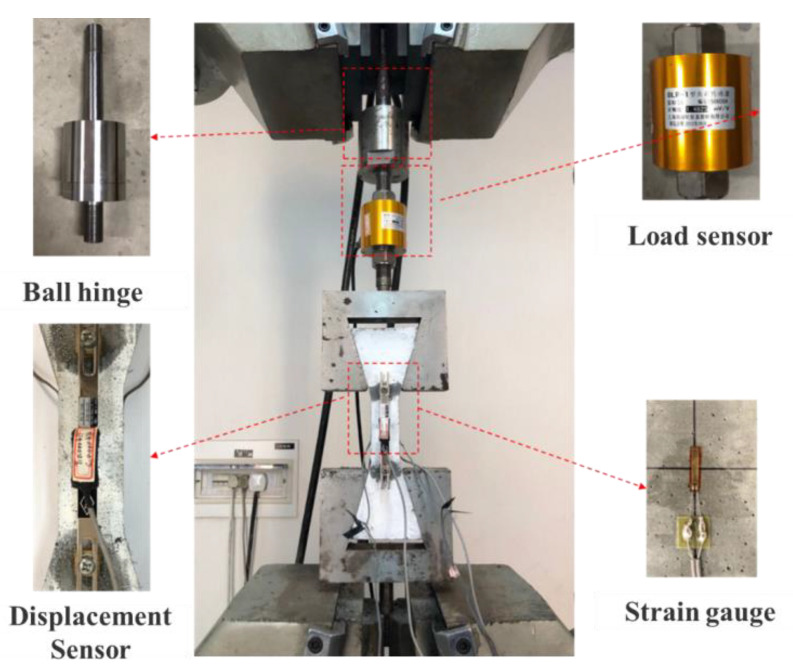
Tensile test set-up.

Dog-bone specimens were fixed in the servo-hydraulic closed-loop test machine using the external clamp, as shown in Figure 4. In order to eliminate the eccentricity of the load, the upper part of the device was equipped with a ball hinge, and the specimen was fixed by two steel mold clamps. The loading rate was 0.2 mm/min. The load sensor was connected between the ball hinge and the external clamp to measure the tensile stress. The average tensile strain was measured by the square copper blocks and the screw cap-fixed displacement sensors on the front and back sides of the specimens. The strain gauges were pasted on the left and right sides to measure the strain of the specimens during the testing. 

## 3. Results and Discussion

### 3.1. Compressive Strength

**Figure 5:**
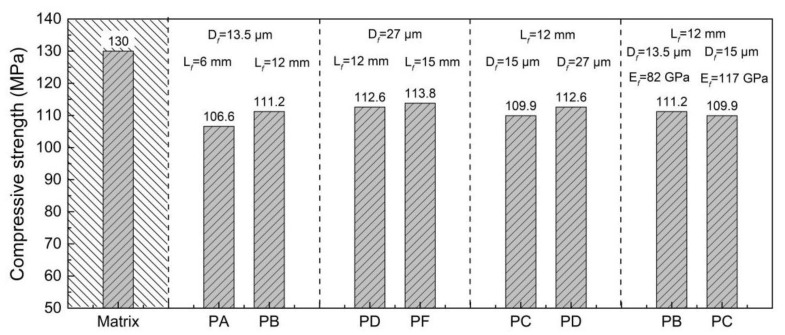
Compressive strength of PE fiber reinforced UHPC.

Figure 5 shows the 28 d compressive strength value of the UHPC with PE fiber. Five kinds of PE fiber were used in this test. *PA* specimen, *PB* specimen, *PC* specimen, *PD* specimen and *PF* specimen represent the UHPC with different types of PE fibers (fiber volume content is 2% vol.). From Figure 5, it can be seen that the compressive strength of the UHPC without fiber is approximately 130 MPa. The addition of PE fibers led to the decrease of compressive strength of the UHPC. The effects of the fiber length, diameter and elastic modulus of the PE fiber on the compressive strength (about 110 MPa) of the UHPC were not significant, and the range of variation was approximately 6.7%. Compared with the other types of PE fibers, the length, diameter and elastic modulus of the *PA* fiber were the minimum; hence, the adhesion between the fiber and the matrix was relatively low. It is easy to produce the transverse deformation of UHPC, causing cracks and a reduction of load-carrying capacity. Therefore, the *PA* specimen exhibited the lowest compressive strength of the specimens. Compared with the compressive strength of the different specimens, the range of each group was *PF* specimen > *PD* specimen > *PB* specimen > *PC* specimen > *PA* specimen. The higher length and diameter of PE fibers were a benefit for the compressive strength of the UHPC, but the influence was not significant.

### 3.2. Tensile Behavior

The uniaxial tensile parameters (first crack stress $\sigma_{fc}$, first crack strain $\varepsilon_{fc}$, ultimate tensile stress $\sigma_{tu}$,  ultimate tensile stress $\varepsilon_{tu}$ and elasticity modulus ${\bar{E}}_{t}$) of the UHPC with PE fibers are shown in Table 5.

**Figure 6:**
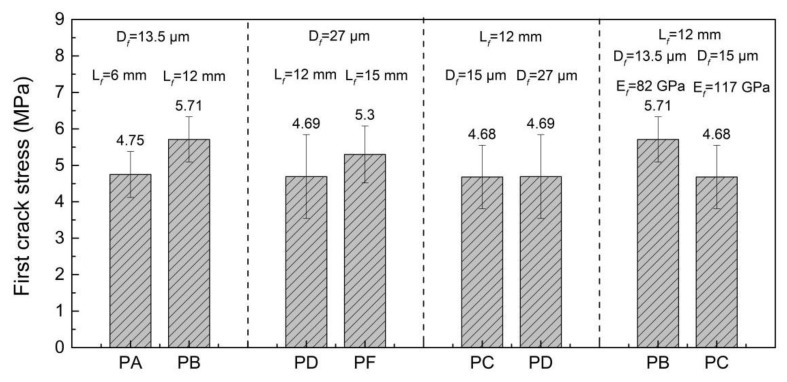
First crack strength of PE fiber-reinforced UHPC.

**Figure 7:**
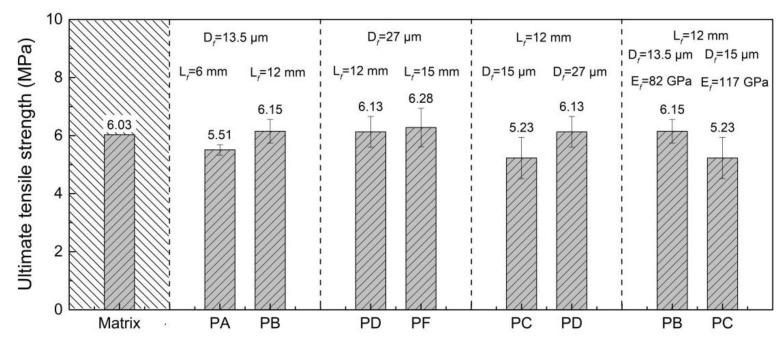
Tensile strength of PE fiber-reinforced UHPC.

Figure 6 and Figure 7 present the $\sigma_{fc}$ and $\sigma_{tu}$ of the UHPC reinforced with different kinds of PE fibers. The tensile experiment on the matrix showed that the tensile strength of the UHPC matrix without PE fibers was 6.03 MPa. Unlike the effect of PE fibers on the compressive strength of UHPC, the incorporation of PE fibers did not always result in a decrease in tensile strength. The tensile strength of the *PD*, *PB* and *PF* specimens was higher than 6 MPa, which was slightly higher than the tensile strength of the matrix without PE fibers. However, the *PA* and *PC* specimens were slightly lower than the matrix. The $\sigma_{fc}$ of the UHPC reinforced with PE fibers was lower than the tensile strength of the matrix, which may be because of the increase of the matrix defects due to the incorporation of the PE fibers. The effects of the length, diameter and elastic modulus of the PE fibers on the $\sigma_{fc}$ and $\sigma_{tu}$ of the UHPC were similar. Higher PE fiber length and fiber diameter lead to the increase of the $\sigma_{fc}$ and $\sigma_{tu}$ of the UHPC, whereas the increase of the fiber elastic modulus exhibited the opposite result. The tensile strength of the *PF* specimen was 20% higher than that of the *PC* specimen, which can be attributed to their higher fiber length (15 mm vs. 12 mm) and diameter (27 mm vs. 15 mm). The influence of the PE fiber types on the tensile strength of the UHPC was more significant than on the compressive strength. 

**Figure 8:**
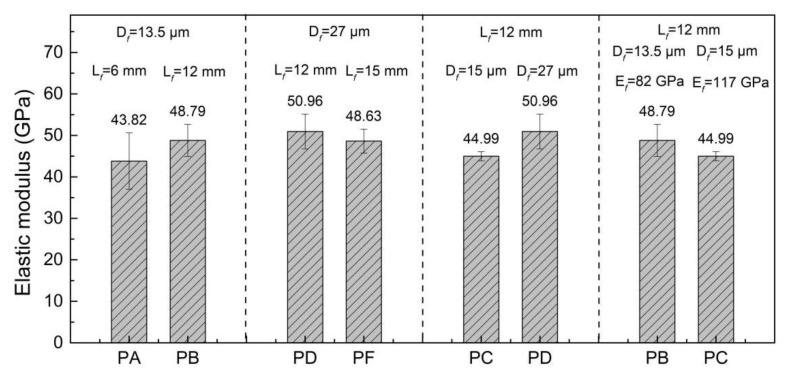
Elastic modulus of PE fiber-reinforced UHPC.

Figure 8 presented the elastic modulus of both UHPCs under investigation. The PD specimen possess the highest ${\bar{E}}_{t}$, while the PA specimen exhibited the lowest ${\bar{E}}_{t}$ (50.15 GPa vs. 42.21 GPa). The increase of the fiber diameter and the decrease of the elastic modulus attributed to the increase of the elastic modulus in the UHPC. The influence of the fiber length on the elastic modulus of the UHPC depended on the fiber diameter. When the fiber diameter was 13.5 mm, the increase in fiber length led to an increase in the ${\bar{E}}_{t}$ of the UHPC. However, when the fiber diameter was 27 mm, the opposite phenomenon was observed. 

Although the tensile strength of PE fiber is similar to that of steel fiber (tensile strength about 3000 MPa), the elastic modulus of PE fiber is lower than that of steel fiber ($\bar{E_{t}}$ of *PD* fiber, *PC* fiber and *PF* fiber is 117 GPa, $\bar{E_{t}}$ of *PA* fiber and *PF* fiber is 85 GPa, $\bar{E_{t}}$ of steel fiber is about 200 GPa [31]). This implies that PE fiber easily causes the deformation and the crack width cannot be restrained well. In addition, the surface of the PE fiber has a low coefficient of friction. This results in the low friction strength between the PE fiber and the matrix. At the same time, the addition of PE fibers increases the interface, and the interface transition zone (ITZ) significantly increases. UHPC easily produces microcracks under loading, and the fiber is easy to pull out or break from the matrix. Therefore, unlike the steel fiber, the addition of PE fiber cannot increase the tensile strength of the UHPC significantly. Moreover, some kinds of PE fiber, such as *PA* and *PC* fibers, may cause a negative effect on the tensile strength of UHPC.

### 3.3. Tensile Strains and Stress Curve

**Figure 9:**
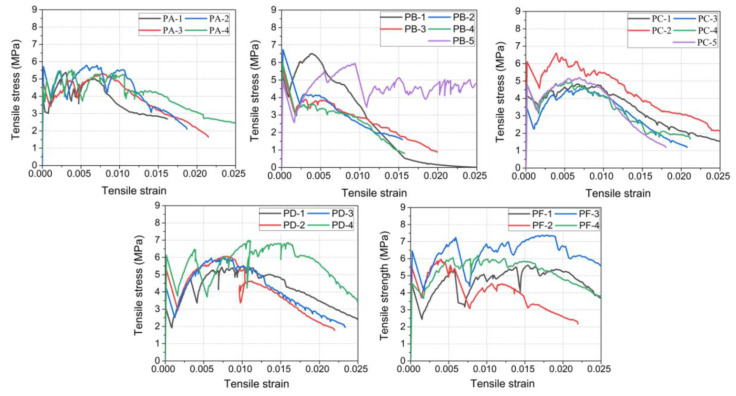
Relationship between tensile stress and strain of UHPC with PE fiber.

**Figure 10:**
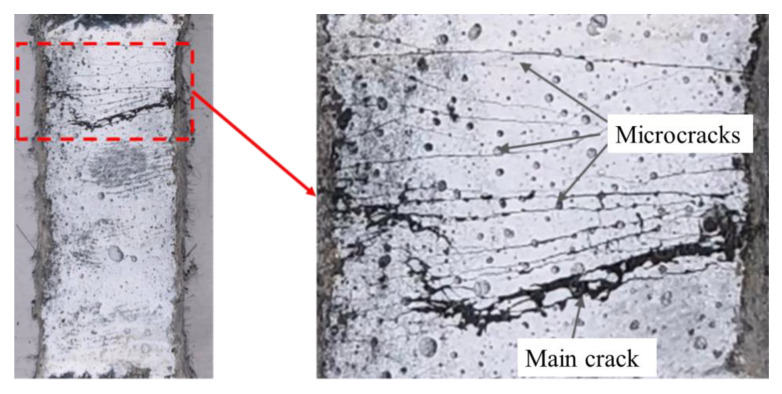
Multi-crack pattern of the *PF* specimen.

Figure 9 shows the tensile stress-strain curve of each specimen. From Figure 9, it can be seen that the fluctuation of the stress-strain curves was obvious, and it was caused by the formation of multiple cracks. Although PE fiber possess a relatively low elastic modulus, such fibers can also provide sufficient crack-bridging strength between cracks due to their smaller fiber diameter. The diameter of PE fiber is 13.5–27 μm, which is less than 13.5% of steel fiber. The approximately thousand fibers in each square centimeter can bridge cracks and prevent deformation localization at the crack site. The bridging force transmitted through the interface between the fiber/matrix can cause the emergence of new cracks. The tensile load-carrying capacity increased continually, and more cracks appeared in the material until the formation of a main crack. Figure 10 shows the multi-crack pattern of the *PF* specimen, where many microcracks appeared around the main crack. The formation of multi-cracks can improve the strain capacity and toughness performance of UHPC after cracking behaviour occurs.

**Figure 11:**
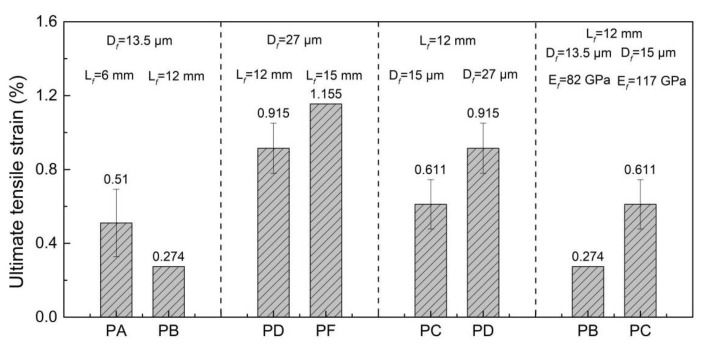
Comparison of the ultimate tensile strain of different specimens.

Figure 11 shows the comparison of the ultimate tensile strain of different specimens. From Figure 11, it can be seen that the geometrical and mechanical parameters of the PE fiber led to the obvious difference in the tensile strain of the different specimens. The ultimate tensile strain of the five groups of specimens was ordered as: *PF* specimen > *PD* specimen > *PC* specimen > *PA* specimen > *PB* specimen. The increase of the fiber diameter and the elastic modulus attributed to the increase of the tensile strain capacity of the UHPC. When the fiber diameter was 27 μm, increasing the fiber length from 12 mm to 15 mm could further improve the tensile capacity of the UHPC. It can be observed that the *PF* fiber exhibited a remarkable effect on the ultimate tensile strain of the UHPC, where the ultimate tensile strain of the *PF* specimen reached 1.155%. 

**Figure 12:**
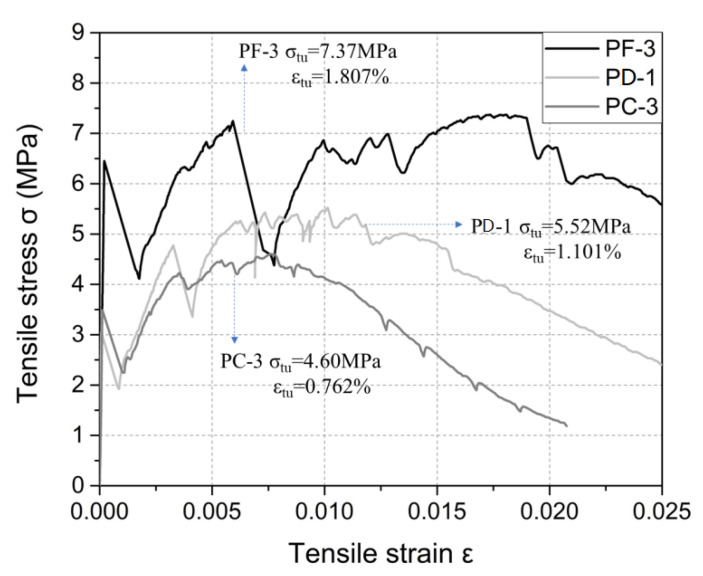
Tensile stress-strain curve of different specimens.

Figure 12 compares the stress-strain curves of the *PC*, *PD* and *PF* specimens. The *PC*-3, *PD*-1 and *PF*-3 specimens were used to compare the influence of different PE fibers on the stress-strain curves. 

From Figure 12, it can be seen that the tensile strength of the *PF*-3 specimen was 7.37 MPa and its tensile strain was 1.807%. Compared with the *PD*-1 specimen, the tensile strength and tensile strain of the *PF*-3 specimen increased by about 33.5% and 63.7%, respectively, and the aspect ratio of the *PF* fiber was 1.25 times that of the *PD* fiber. It can be seen that when the diameter of the PE fibers was the same, increasing the aspect ratio could significantly improve the tensile properties of the UHPC. 

In addition, the tensile strength of the *PC*-3 specimen was 4.60 MPa, and the tensile strain was 0.762%. Compared with the *PD*-1 specimen, the tensile strength and tensile strain of the *PC*-3 specimen decreased by 16.7% and 30.8%, respectively, and the aspect ratio of the *PC* fiber is 2 times that of the *PD* fiber. Therefore, when the fiber length is the same, increasing the aspect ratio may reduce the tensile properties of the UHPC.

From the discussion above, when the fiber diameter is constant, increasing the aspect ratio of PE fiber increases its length. The longer PE fiber can restrain cracks more effectively, resulting in the enhanced tensile strength and tensile strain of the UHPC. When the fiber length is constant, increasing the aspect ratio of PE fiber decreases its diameter. Because the diameter of PE fiber is micron-level, the fiber is easy to ball, especially with the small diameter. At the same fiber volume content, the number of fibers with smaller diameter is enhanced, which maybe increase the difficulty of fiber dispersion and lead to fiber balling in the UHPC with a small water-to-cement ratio. Non-uniform fiber dispersion may lead to a decrease in tensile properties, thus a suitable water-to-cement ratio could improve the fluidity and the quality of the UHPC, and it could significantly increase the mechanical properties of the UHPC with PE fiber.

### 3.4. Tensile Toughness

The matrix of the UHPC is a kind of highly brittle material. In order to overcome the brittle characteristic of the composite, PE fiber was chosen for addition to the UHPC matrix. As the fibers were randomly distributed in the matrix, when the specimen was cracked, the crack tip close to the fibers was forced to change direction and formed some finer cracks. When the microcrack developed into a macrocrack, the fibers bridged the cracks and delayed the cracking of the matrix, which significantly improved the energy absorption capacity of the matrix.

**Figure 13:**
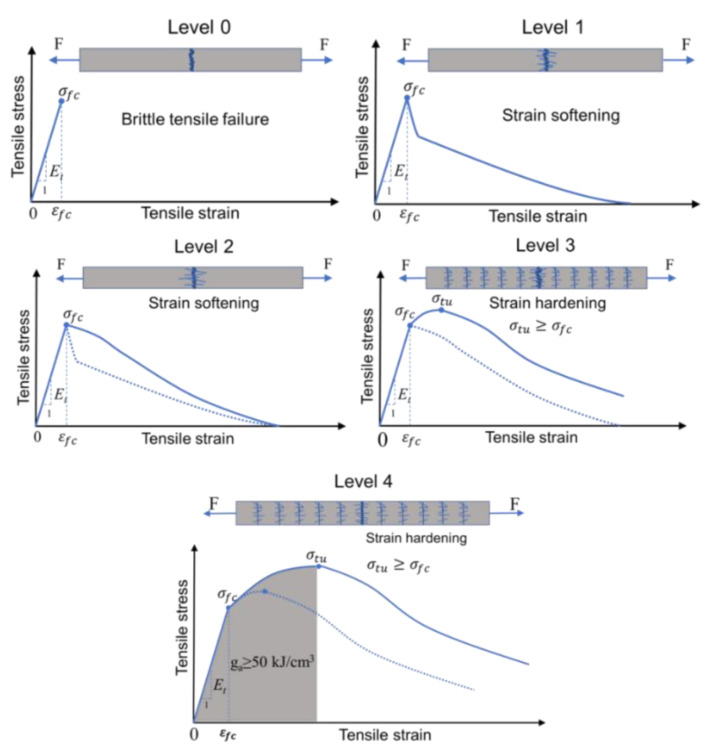
Evaluation of tensile performance grade of UHPC [32].

There is still no set of systematic and comprehensive evaluation methods regarding the tensile toughness of UHPC. According to the performance grading scheme proposed by Naaman and Reinhardt [32], as shown in Figure 13, the tensile properties of UHPC can be divided into the five levels: Level 0 was used to illustrate the tensile behavior of plain UHPC without fibers as a control group. The tensile stress-strain curve has only an elastic phase, and the matrix cracks and fails after reaching the peak load. With the addition of fibers, the tensile behavior of the UHPC can be improved due to the fiber bridging effect. As illustrated in Level 1, the tensile stress-strain curve of the UHPC exhibits the stress-softening section. In Level 2, the softening section of the tensile stress-strain curve is more obvious, and the energy absorption capacity of the composite is improved. However, the cracking formed in the composite is still the single crack. In Level 3, the composite exhibits strain-hardening behavior under tensile loading. The cracking mode of the composite changes from the single crack to the multiple crack pattern. The ultimate tensile strain of the specimen is greatly improved. In Level 4, the composite exhibits higher ultimate tensile strength and ultimate tensile strain. The strain-hardening section of the composite becomes more obvious, and the energy absorption capacity greatly improves. 

**Figure 14:**
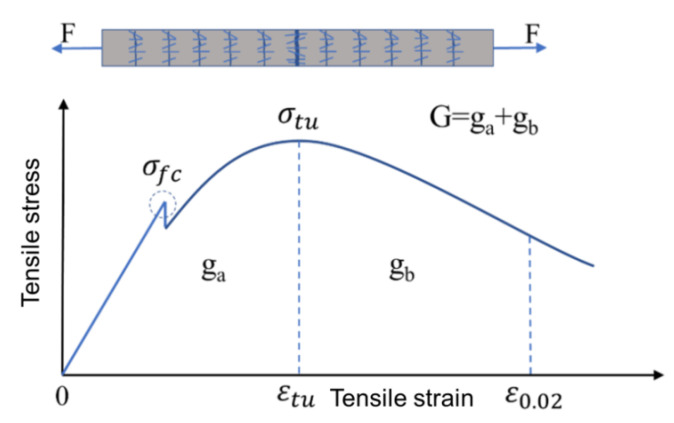
Schematic diagram of the energy absorption capacity of UHPC.

Based on the experiment results, the tensile properties of all the types of PE fiber-reinforced UHPC in this study satisfied performance Level 3. Figure 14 shows the schematic diagram of the energy absorption capacity of the UHPC, where $g_{a}$ represents the energy absorption capacity of the substrate prior to the softening section. That was derived from the tensile stress-strain curve of the specimen as a definite integral, which was expressed on the curve image as the area of the curve with the X-envelope. The calculation formula is expressed in Equation (1). According to the Wille’s suggestion, a value of $g_{a}$ higher than 50 kJ/m^3^ is classified as high-toughness UHPC (Level 4) [33].

(1) $$g_{a}=\int_{0}^{\varepsilon_{tu}}\sigma (\varepsilon )d\varepsilon$$

where $\varepsilon_{tu}$ is ultimate tensile strain.

In order to quantify the energy absorption capacity of the UHPC during the total stretching process, $g_{b}$ and *G* were introduced. $g_{b}$ was defined as the energy absorption capacity of the softening section of the UHPC. The energy of the softening section $g_{b}$ was calculated in the interval from $\varepsilon_{tu}$ to ε = 0.020. When the tensile strain reached 0.020, the tensile stress generally decreased to below 50% of the peak stress. Most of the fibers failed, and the residual energy of the UHPC was less. $g_{b}$ can be expressed in Equation (2).

(2) $$g_{b}=\int_{\varepsilon_{tu}}^{\varepsilon =0.02}\sigma (\varepsilon )d\varepsilon$$

*G* is the sum of $g_{a}$ and $g_{b}$, representing the energy absorption capacity of the tensile process.

(3) $$G=g_{a}+g_{b}$$

**Figure 15:**
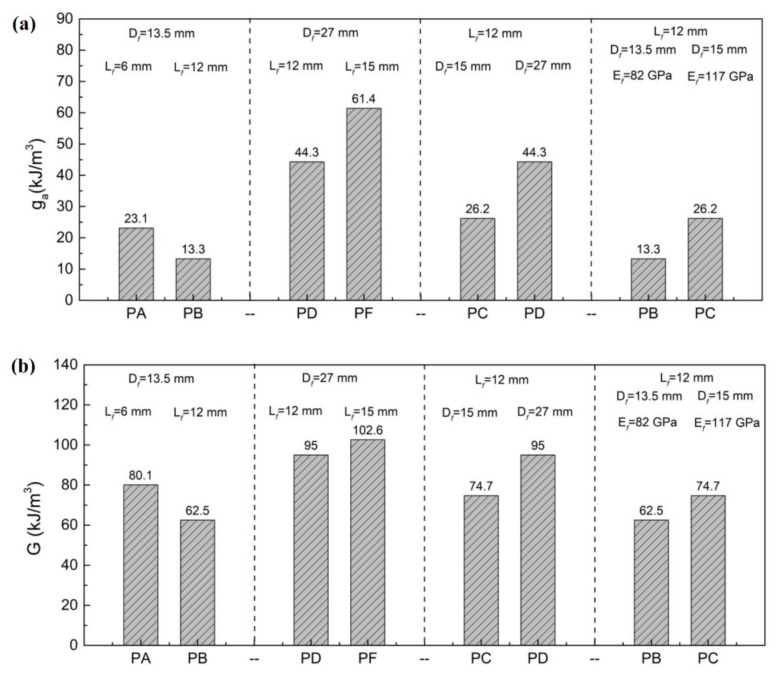
(**a**) Energy absorption capacity of UHPC with PE fiber before strain softening; (**b**) Overall energy absorption capacity of UHPC with PE fiber.

Figure 15 shows the energy absorption capacity of the UHPC with different types of PE fiber before strain softening and the whole process. As shown in Figure 15a, the energy absorption capacity of each group before the softening section was ordered as: *PF* specimen > *PD* specimen > *PC* specimen > *PA* specimen > *PB* specimen. The tensile toughness of the *PF* specimen was the highest among the five groups, with a *g_a_* of 61.37 kJ/m³, which could be classified as the tensile performance grade of Level 4. Although the tensile toughness of the *PD* specimen was not the highest, its *g_a_* also reached 44.30 kJ/m³ and showed a high tensile toughness. Comparison of the *g_a_* of the *PD* specimen and the *PF* specimen in Figure 15 indicates that the tensile toughness of the UHPC was enhanced by the higher length of the PE fiber. However, when the fiber diameter was 13.5 μm, the increase in fiber length did not lead to an increase in the *g_a_* of the UHPC. *PB* fiber possessed the highest aspect ratio but the lowest diameter, which led to the uneven fiber dispersion during the mixing process. The tensile toughness of the *PB* specimen was the lowest where some specimens lost the strain-hardening section in the tensile curve and the tensile strain was low. The result in Figure 15 also shows that the *g_a_* of the UHPC became enhanced with the increase of the fiber diameter and the elastic modulus. The overall energy absorption capacity *G* of each group in Figure 15b above reflected similar results. Therefore, the diameter of the PE fiber was the main factor affecting the energy consumption of the UHPC. Higher fiber diameter, fiber length and elastic modulus were benefits for the *g_a_*. 

## 4. Conclusions

(1) High fiber length and fiber diameter of PE fibers are beneficial for the compressive strength and tensile strength of UHPC. The influence of PE fiber types on the tensile strength of UHPC was more significant than those on compressive strength. 

(2) The increase of fiber diameter and elastic modulus contributed remarkably to the increase of the tensile strain capacity of the UHPC. PF fiber (Fiber length: 15 mm; Fiber diameter: 27 μm; Elastic Modulus: 117 GPa) exhibits a remarkable effect on the ultimate tensile strain of the UHPC, where the ultimate tensile strain of the PF specimen reached 1.155 %.

(3) The overall energy absorption capacity G and the energy absorption capacity of the substrate prior to the softening section ga of the UHPC enhanced with the increase of the fiber diameter and the elastic modulus. The diameter of the PE fiber is the main factor affecting the energy consumption of the UHPC.

(4) PE fiber increased the strain capacity and toughness performance of the UHPC under tensile loading. The multiple crack pattern of the UHPC was observed under the tensile loading due to the fiber bridging effect. Among the five types of PE fiber, the PF fiber was the optimum PE fiber to increase the tensile mechanical behavior of the UHPC.

## Figures and Tables

**Table 1:** Basic parameters of cement.

Loss on Ignition (%)	Sulfur Trioxide (%)	Magnesium Oxide (%)	Chloride Ion (%)	Cement Standard Consistency (%)	Specific Surface Area (m²/kg)	28 d Flexural Strength (MPa)	28 d Compressive Strength (MPa)
1.98	2.21	1.98	0.013	28.2	397	8.3	53.7

**Table 2:** Chemical composition and physical properties of silica fume.

Chemical Composition			Loss on Ignition (%)	Water Content (%)	45 μm Sieve Surplus (%)
SiO_2_ ≥ 87.2	K_2_O ≤ 0.86	Na_2_O ≤ 0.13	≤3.63	0.77	1.61

**Table 3:** Mixing ratio of basic matrix (kg/m^3^).

Cement	Silica Fume	Slag	Sand	Water Reducer	Defoamer	Water
1000	200	100	1200	20	3	240

**Table 4:** Morphology and basic parameters of PE fiber.

PE Fiber	Length (mm)	Diameter (μm)	Elastic Modulus (GPa)	Tensile Strength (MPa)	Slump of Matrix with 2% vol. Fiber (mm)
*PA* fiber	6	13.5	82	2640	141
*PB* fiber	12	13.5	82	2640	137
*PC* fiber	12	15	117	3312	133
*PD* fiber	12	27	117	2976	142
*PF* fiber	15	27	117	2976	153

**Table 5:** Uniaxial tensile parameters of UHPC with PE fiber.

Specimen	$\sigma_{fc}$ (MPa)	${\bar{\sigma }}_{fc}+s\;(\mathrm{MPa})$	$\varepsilon_{fc}$ (%)	${\bar{\varepsilon }}_{fc}+s$ (%)	$\sigma_{tu}\;(\mathrm{MPa})$	${\bar{\sigma }}_{tu}+s\;(\mathrm{MPa})$	$\varepsilon_{tu}$ (%)	${\bar{\varepsilon }}_{tu}+s$ (%)	$E_{t}$ (GPa)	${\bar{E}}_{t}+s$ (GPa)
*PA*-1	3.97	4.75 ± 0.63	0.006	0.0093 ± 0.0034	5.38	5.51 ± 0.18	0.305	0.5095 ± 0.183	37.88	42.21 ± 6.76
*PA*-2	4.76		0.011		5.32		0.778		50.00	
*PA*-3	5.72		0.014		5.79		0.572		38.76	
*PA*-4	4.55		0.006		5.53		0.383		--	
*PB*-1	5.34	5.71 ± 0.62	0.011	0.0140 ± 0.002	6.51	6.15 ± 0.41	0.383	0.2736 ± 0.362	47.17	48.79 ± 3.92
*PB*-2	5.64		0.013		5.64		0.013		44.25	
*PB*-3	6.74		0.017		6.74		0.017		49.50	
*PB*-4	5.94		0.015		5.94		0.015		54.90	
*PB*-5	4.89		0.014		5.94		0.94		48.08	
*PC*-1	4.15	4.68 ± 0.87	0.015	0.0152 ± 0.0019	4.82	5.23 ± 0.71	0.684	0.6114 ± 0.134	48.54	47.34 ± 1.13
*PC*-2	6.11		0.016		6.6		0.395		47.17	
*PC*-3	3.49		0.012		4.6		0.762		46.30	
*PC*-4	4.83		0.015		4.95		0.52		--	
*PC*-5	4.8		0.018		5.19		0.696		--	
*PD*-1	3.06	4.69 ± 1.15	0.007	0.0125 ± 0.0035	5.52	6.13 ± 0.53	1.014	0.9148 ± 0.136	47.17	50.15 ± 4.24
*PD*-2	5.29		0.015		6.05		0.806		45.87	
*PD*-3	4.27		0.012		5.97		0.758		54.35	
*PD*-4	6.13		0.016		6.99		1.081		53.19	
*PF*-1	4.59	5.30 ± 0.78	0.014	0.0153 ± 0.0044	5.61	6.28 ± 0.66	1.536	1.1550 ± 0.553	48.08	48.63 ± 2.86
*PF*-2	5.58		0.009		5.96		0.396		49.50	
*PF*-3	6.45		0.021		7.37		1.807		45.05	
*PF*-4	4.59		0.017		6.18		0.881		51.89	

## References

[1] Qin J., Dai F., Ma H., Dai X., Li Z., Jia X., Qian J. (2022). Development and characterization of magnesium phosphate cement based ultra-high performance concrete. Compos. Part B-Eng..

[2] Yoo D., Banthia N. (2016). Mechanical properties of ultra-high-performance fiber-reinforced concrete: A review. Cem. Concr. Compos..

[3] Gong J., Ma Y., Fu J., Hu J., Ouyang X., Zhang Z., Wang H. (2022). Utilization of fibers in ultra-high performance concrete: A review. Compos. Part B-Eng..

[4] Schmidt M., Fehling E. (2005). Ultra-high-performance concrete: Research, development and application in Europe. ACI Spec. Publ..

[5] Xu S., Wu P., Li Q., Zhou F., Chen B. (2021). Experimental investigation and numerical simulation on the blast resistance of reactive powder concrete subjected to blast by embedded explosive. Cem. Concr. Compos..

[6] Xu S., Zhou F., Li Q., Wu P. (2021). A novel dynamic cavity expansion model to predict the resistance of reactive powder concrete (RPC) against projectile impact. Compos. Part B-Eng..

[7] Zhang W., Zhang Y., Zhang G. (2012). Static, dynamic mechanical properties and microstructure characteristics of ultra-high performance cementitious composites. Sci. Eng. Compos. Mater..

[8] Yoo D., Kim S., Park G.J., Park J.J., Kim S.W. (2017). Effects of fiber shape, aspect ratio, and volume fraction on flexural behavior of ultra-high-performance fiber-reinforced cement composites. Compos. Struct..

[9] Xu L., Wu F., Chi Y., Cheng P., Zeng Y., Chen Q. (2019). Effects of coarse aggregate and steel fibre contents on mechanical properties of high performance concrete. Constr. Build. Mater..

[10] Yu R., Spiesz P., Brouwers H. (2014). Mix design and properties assessment of Ultra-High Performance Fibre Reinforced Concrete (UHPFRC). Cem. Concr. Res..

[11] Yoo D., Kim S., Kim J.J., Chun B. (2019). An experimental study on pullout and tensile behavior of ultra-high-performance concrete reinforced with various steel fibers. Constr. Build. Mater..

[12] Wu Z., Shi C., He W., Wu L. (2016). Effects of steel fiber content and shape on mechanical properties of ultra high performance concrete. Constr. Build. Mater..

[13] Wu Z.M., Shi C.J., Khayat K.H. (2019). Investigation of mechanical properties and shrinkage of ultra-high performance concrete: Influence of steel fiber content and shape. Compos. Part B-Eng..

[14] Liu J., Han F., Cui G., Zhang Q., Jin L. (2016). Combined effect of coarse aggregate and fiber on tensile behavior of ultra-high performance concrete. Constr. Build. Mater..

[15] Yoo D., Kim M., Kim S.W., Park J.J. (2017). Development of cost effective ultra-high-performance fiber-reinforced concrete using single and hybrid steel fibers. Constr. Build. Mater..

[16] Yoo D., Kang S., Yoon Y. (2016). Enhancing the flexural performance of ultra-high-performance concrete using long steel fibers. Compos. Struct..

[17] Dong J.K., Park S.H., Ryu G.S., Koh K.T. (2011). Comparative flexural behavior of Hybrid Ultra High Performance Fiber Reinforced Concrete with different macro fibers. Constr. Build. Mater..

[18] Yoo D., Yoon Y. (2015). Structural performance of ultra-high-performance concrete beams with different steel fibers. Eng. Struct..

[19] Huang B.T., Wu J., Yu J., Dai J.G., Li V.C. (2021). Seawater sea-sand engineered/strain-hardening cementitious composites (ECC/SHCC): Assessment and modeling of crack characteristics. Cem. Concr. Res..

[20] Yu K., Hou M., Zhu H., Li V.C. (2021). Self-healing of PE-fiber reinforced lightweight high-strength engineered cementitious composite. Cem. Concr. Compos..

[21] Zhao X., Li Q., Xu S. (2020). Contribution of steel fiber on the dynamic tensile properties of hybrid fiber ultra high toughness cementations composites using brazilian test. Constr. Build. Mater..

[22] Huang B., Weng K., Zhu J., Xiang Y., Dai J., Li V.C. (2021). Engineered/strain—Hardening cementitious composites (ECC/SHCC) with an ltra-high compressive strength over 210 MPa. Comp. Comm..

[23] Ranade R., Li V.C., Heard W.F. (2015). Tensile rate effects in high strength-high ductility concrete. Cem. Concr. Res..

[24] Yu K.-Q., Dai J.-G., Lu Z.-D., Poon C.-S. (2018). Rate-dependent tensile properties of ultra-high performance engineered cementitious composites (UHP-ECC). Cem. Concr. Compos..

[25] Yu K., Ding Y., Zhang Y.X. (2020). Size effects on tensile properties and compressive strength of engineered cementitious composites. Cem. Concr. Compos..

[26] Zhang D., Tu H., Li Y., Weng Y. (2022). Effect of fiber content and fiber length on the dynamic compressive properties of strain-hardening ultra-high performance concrete. Constr. Build. Mater..

[27] Yu K.Q., Yu J.T., Dai J.G., Lu Z.D., Shah S.P. (2018). Development of ultra-high performance engineered cementitious composites using polyethylene (PE) fibers. Constr. Build. Mater..

[28] He S., Qiu J., Li J., Yang E. (2017). Strain hardening ultra-high performance concrete (SHUHPC) incorporating CNF-coated polyethylene fibers. Cem. Concr. Res..

[29] Hannawi K., Bian H., Prince-Agbodjan W., Raghavan B. (2016). Effect of different types of fibers on the microstructure and the mechanical behavior of Ultra-High Performance Fiber-Reinforced Concretes. Compos. Part B-Eng..

[30] Oh T., You I., Banthia N., Yoo D.Y. (2021). Deposition of nanosilica particles on fiber surface for improving interfacial bond and tensile performances of ultra-high-performance fiber-reinforced concrete. Compos. Part B-Eng..

[31] Ding Y., Ning X., Zhang Y., Pacheco-Torgal F., Aguiar J.B. (2014). Fibres for enhancing of the bond capacity between gfrp rebar and concrete. Constr. Build. Mater..

[32] Naaman A.E., Reinhardt H.W. Setting the stage: Toward performance based classification of FRC composites. Proceedings of the 4th RILEM Symposium on High Performance Fiber Reinforced Cement, Composites (HPFRCC4).

[33] Wille K., El-Tawil S., Naaman A.E. (2014). Properties of strain hardening ultra high performance fiber reinforced concrete (UHP-FRC) under direct tensile loading. Cem. Concr. Compos..

